# A snapshot of drug background levels on surfaces in a forensic laboratory

**DOI:** 10.1016/j.forc.2018.09.001

**Published:** 2018

**Authors:** Edward Sisco, Marcela Najarro, Amber Burns

**Affiliations:** a National Institute of Standards and Technology, Gaithersburg, MD 20899, United States; b Maryland State Police, Forensic Science Division, Pikesville, MD 21208, United States

**Keywords:** Narcotics, Background, Quantitation, LC/MS/MS, TD-DART-MS

## Abstract

While background studies have been commonplace in many occupational fields for a long time, attempts to understand the chemical background in forensics labs has been largely understudied. Such studies can help define the efficiency of cleaning procedures and the integrity of collected data, which is becoming increasingly important due to improving sensitivity of instrumentation and the prevalence with which potent drugs of abuse, such as the opioids, are being seen. The results from this study provide a snapshot of the drug background levels on surfaces in a laboratory system comprised of a central laboratory and two satellite laboratories. Samples were collected from work surfaces by swiping with meta-aramid wipes, and extracted for analysis by LC/MS/MS, for quantitation, and TD-DART-MS, for non-targeted screening. Surfaces were sampled from within the drug unit (where drug evidence is processed) and the evidence receiving unit (where drug cases are handled) in all laboratories as well as the report writing area, the toxicology unit and the crime scene unit in the central laboratory. Results showed that the background was restricted primarily to the benches, balances, and instrumentation within the drug unit – with approximately an order of magnitude higher concentrations observed on the balances, compared to the benches. Higher levels were also observed in analyst specific surfaces when compared to general use surfaces within the drug unit – which corresponded to where bulk evidence handling was completed. Background in the evidence receiving and report writing sections was minimal. Comparison of the main laboratory to the satellite laboratories showed similarities amongst frequently encountered drugs like cocaine, but noticeable differences in opioids which could be attributed to differences in the make-up of exhibits each laboratory receives. Understanding the background levels of drugs in a forensic laboratory environment is crucial to improving cleaning protocols, helping define detection limits for highly sensitive analyses, and providing additional results to the broader community that has been establishing background levels in other environments.

## 1. Introduction

Characterizing the chemical background of an operational environment is a common practice in a number of occupational fields, from environmental [1–3] to pharmaceutical [4–6] to electronics manufacturing [7]. These and other fields attempt to understand and quantify the background of compounds of interest for many reasons, including occupational or public health [8], quality control [9], and remediation [10]. Background sampling can be performed by a number of different methods including surface sampling [8,11] done by wiping a surface with a collection wipe, air sampling [12], or water sampling [13].

To help establish a baseline background level, it is important to understand what the background is comprised of and how much there is. A non-targeted screening analysis is a useful tool to identify what compounds of interest might be present. Following this with a targeted quantitative analysis can provide both confirmation of the compounds identified and their concentration or mass per unit area. Once this baseline is established, repeated sampling can help answer questions relating to persistence or temporal changes. If mitigation strategies are implemented, routine sampling can also be used to evaluate the effectiveness of these processes.

Several studies have investigated the levels of illicit drugs in various environments. Most of these studies have focused on detection of drugs in waste waters and surface water for a variety of purposes – from understanding the health effects [14,15] to estimating drug usage [16] to examining the potential investigative value of such an analysis [17]. In addition to wastewater and surface water, work has been completed to measure the level of illicit drugs in the air of cities across the world [18–20]. This type of analysis has shown that pg m^−3^ to ng m^−3^ levels of cocaine were observed in most cities while heroin and cannabinol could be detected less frequently [19].

Given that illicit drugs can be detected in the air, it is reasonable to assume that environmental surface background also exists – either as residual powder or as condensed aerosols from smoking. Work by Smith and McGrath [21] looked at detection of cocaine off of surfaces that people contact on a daily basis (*i.e.* fuel pumps, shopping carts, and door handles) and found that upwards of 75% off all the surfaces tested were found to contain detectable levels of cocaine. While no attempts were made to quantify the level of cocaine off of these surfaces, Jenkins [22] investigated the level of cocaine on US currency and found that levels can, at times, exceed 1 mg bill^−1^, but were commonly in the range of tens to hundreds of micrograms per bill. Other work has investigated the surface levels of methamphetamine in clandestine laboratories and found hundreds of micrograms per square meter [23]. Removal efficiencies off of household surfaces has also been studied [24].

While surface levels of drugs in forensic laboratories has not been discussed in the literature, this question has been examined in the context of police stations. Doran et al. have completed substantial studies investigating the levels of drugs, and persistence of those drugs, in police stations throughout Australia [25,26]. The work by Doran et al. highlighted increased prevalence of illicit drugs in police stations, relative to public spaces, which was attributed to the handling of drug evidence at the stations. In most instances, the level of drugs detected was low (< 50 ng) but several surfaces did contain micrograms of material. Given that forensic laboratories also handle bulk amounts of illicit drugs on a regular basis, it is reasonable to assume that handling of drug evidence will potentially contribute to an elevated background level, compared to public spaces. Opening and handling of bulk quantities of drugs can lead to aerosolized release of this material, typically in the form of particulate, throughout the laboratory. Like any other particulate trace, there is a reasonable expectation that this residue will be transferred throughout the laboratory via touch, direct transfer, and/ or suspension of particulate in the air.

Additionally, forensic laboratories are currently being faced with increasing backlogs [27,28] and decreasing budgets to tackle such backlogs. This dichotomy is especially significant in drug chemistry units which are constantly being presented with new and increasingly potent compounds (*i.e.* fentanyl analogs). To tackle this issue, laboratories are implementing, or considering implementing, analytical tools [29] (such as direct analysis in real time mass spectrometry, DART-MS [30–32]) that allow for rapid screening, presumptive testing, and/or triaging. Because these analytical tools typically employ high throughput analysis with minimal to no sample preparation it is crucial to understand background levels of analytes of interest to minimize the likelihood of reporting a false detection. Additionally, as emerging analytical instrumentation becomes increasingly sensitive [31,32], the background level of the chemicals of interest in the analysis chain must be considered. Establishing background levels of compounds of interest in a forensic laboratory can provide drug analysts and laboratory quality managers with valuable information to make informed decisions on a range of topics such as workflow processes, adequate personal protective equipment (PPE), cleaning protocols, and occupational safety hazards.

This study provides a snapshot of the drug background levels in a three-laboratory system (a central laboratory and two satellite laboratories) in order to get a rough understanding of what expected drug background levels may be. Interpretation of these levels from a data quality and occupational health perspective are the focus of ongoing collaborative work. Wipe samples were collected across the drug chemistry unit, evidence receiving unit, toxicology unit, crime scene unit, and report writing section of the central laboratory as well as the drug chemistry unit and evidence receiving of the two satellite laboratories. Samples were analyzed using liquid chromatography-tandem mass spectrometry (LC/MS/MS) for quantitation of 18 drugs and thermal desorption direct analysis in real time mass spectrometry (TD-DART-MS) for non-targeted screening analysis. A total of 60 samples were measured from the central laboratory and an additional 50 samples from the two satellite labs. Surface concentrations of drugs were highest and most diverse within the drug unit, where a total of 15 of the 18 targeted drugs were detected at concentration ranges from 1 pg cm^−2^ to 97 ng cm^−2^. Within the drug unit, balances were found to contain the highest surface concentrations that were typically close to an order of magnitude higher than the benches. Levels observed in the evidence receiving and other units were substantially lower than within the drug unit, and some noticeable differences were observed between the drug units across the three laboratories.

## 2. Materials and methods

### 2.1. Sample collection and extraction

Samples were collected from various locations throughout the laboratory, targeting both areas common to the typical workflow for the analysis of drug evidence and areas where drug cases are not analyzed. At all three laboratories samples were collected from the drug unit and the evidence receiving unit. In addition, samples were also collected from the report writing area (for drug analysts), toxicology unit, and crime scene unit at the central laboratory. Within the drug unit, samples were taken from both general-use surfaces/items, such as chemical hoods and instruments, and analyst-specific surfaces/items, such as balances and benches assigned to individual analysts to process their casework. Additionally, surfaces/items such as benches, storage bins, and door handles in the other units were sampled. All surfaces that were sampled were non-porous. For benches and hoods, the entirety of the surface was sampled (surface area was not controlled but was measured). For balances, the enclosure (pan and surrounding area) in addition to the control panel were sampled. A total of 60 samples were collected from the central laboratory with the majority of the samples collected from the drug unit. An additional 50 samples were collected from the two satellite labs and focused solely on the drug and evidence receiving units. The entirety of the surface was sampled with a single wipe and the surface area determined by photographing the surface sampled and calculating the area using Adobe (San Jose, CA, USA).

Samples were collected with meta-aramid wipes (DSA Detection, North Andover, MA), which are dry wipes commonly used for particle collection in trace contraband detection. The particle collection efficiency of this material off non-porous surfaces has been previously measured at approximately 30% collection, demonstrating that it was an adequate substrate for the collection of trace residues off a variety of surfaces [33]. Potential differences in collection efficiency within the surfaces was not accounted for in the measurement. Samples were collected on the top half of the wipe by wiping in a unilateral direction using two to three fingers to apply firm force (7 N–10 N) and help guide the maximum collection of trace residues into the desired area of the wipe [34]. In addition to sample collection, several wipe blanks were analyzed from the same lot of meta-aramid used for the experiment. Samples were labeled, stored individually in manila envelopes, and transported back to the NIST laboratory for chemical analysis. Any potential loss of collected material due to storage conditions was not studied.

**Fig. 1:**
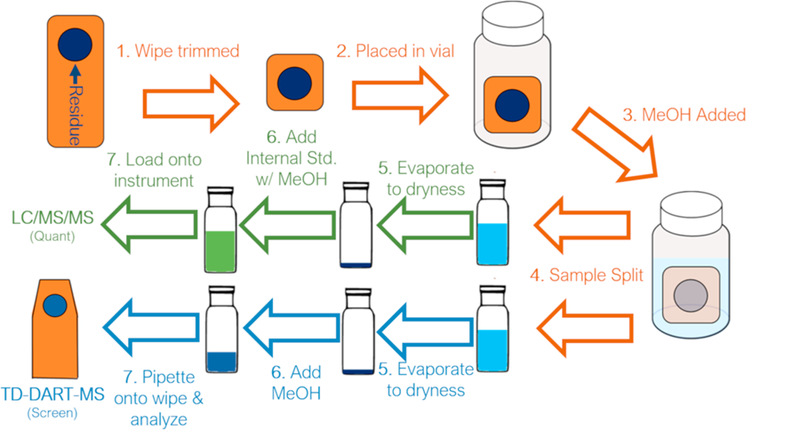
Schematic of the sample preparation and extraction procedures.

Prior to analysis, the wipes were trimmed in size to remove the unused bottom half. The trimmed wipe was then placed in a 10 mL amber glass vial and extracted with 4.0 mL of methanol (Chromasolv Grade, Sigma-Aldrich, St. Louis, MO) by vortexing for 30 s at 3000 rpm. Two milliliters of the extract were quantitatively transferred to an glass vial for the quantitative LC/MS/MS analysis while the remainder was transferred to a second glass vial for screening analysis by TD-DART-MS. Both aliquots were evaporated to dryness under a stream of zero-air nitrogen. The aliquot for quantitation was reconstituted in 500 µL of methanol containing five internal standards while the aliquot for screening analysis was reconstituted in 200 µL of methanol – 10 µL of which was pipetted onto a PTFE-coated fiberglass wipe (DSA Detection, North Andover, MA) for analysis by TD-DART-MS. The vial containing the quantitation aliquot was directly loaded onto the LC/MS/MS system. Fig. 1 depicts the sample preparation procedure.

### 2.2. Chemicals

Standards for the screening and quantitation studies were obtained from either Cayman Chemical (Ann Arbor, MI), Cerilliant (Round Rock, TX), or Sigma-Aldrich (St. Louis, MO) as 1 mg mL^−1^ standards in methanol or acetonitrile (when possible) or as pure crystalline material. Chromasolv-grade methanol (Sigma-Aldrich) was used for sample extraction, reconstitution, and creation of calibration curves. For quantitation, five deuterated internal standards were used: methamphetamine-D_5_, heroin-D_9_, cocaine-D_3_, fentanyl-D_5_, and THC-D_9_ (Cayman Chemical/Cerilliant, 1 mg mL^−1^ solutions). A 1 mL aliquot of each of the internal standards was added to 1 L of methanol, providing an extraction solvent with an internal standard concentration of approximately 1 µg mL^−1^. Chromasolv-grade methanol and water (Sigma-Aldrich) were used for the mobile phase with the addition of formic acid (0.1% v/v).

### 2.3. Quantitation of drugs by LC/MS/MS

LC/MS/MS was chosen for the quantitation of samples due to its increased sensitivity compared to commonly employed gas chromatography mass spectrometry (GC/MS) systems. The system was operated in multiple reaction monitoring (MRM) mode for all analyses and consisted of a Thermo Ulti-Mate 3000 LC system coupled to an Sciex Q-Trap 4000 mass spectrometer. Separation was achieved using a Restek (Bellefonte, PA) Raptor Biphenyl column (150 mm × 4.6 mm × 2.7 µm). The total analysis time was 15 min with a flow rate of 0.75 mL min^−1^ and an injection volume of 10 µL. During the run, a 12-min solvent gradient was used (95% water/5% methanol with 0.1% formic acid to 100% methanol with 0.1% formic acid) followed by a 3-minute isocratic period (100% methanol + 0.1% formic acid). Zero-air nitrogen was used for both the desolvating and nebulizing gases within the electrospray ionization (ESI) source of the MS. Additional ESI source parameters included an operating temperature of 550 °C and a spray voltage of +5500 V. A timed MRM was used to monitor two transitions for all 18 drugs (one transition for quantitation and one transition for confirmatory identification) in addition to one transition for each of the five internal standards. The MRM detection window was set to 60 s and the target scan time was set to 0.1 s.

Table 1 shows the drugs, retention times, quantitative transitions, confirmatory transitions, limits of quantitation – defined as the lowest calibration level run within the linear range – and the internal standard used for each of the 18 drugs investigated. The amount recovered off the wipe was calculated by taking the ratio of the peak areas of a drug to the appropriate internal standard and comparing that ratio to a gravimetrically prepared 13-point calibration curve. Each run on the LC/MS/MS system contained methanol blanks, internal standard blanks, and multiple calibration curve verification (CCV) samples. Additionally, a methanol blank was run in between every injection to minimize potential carry-over between measurements. From the amount recovered off the wipe, and a measurement of the surface area sampled, a surface area concentration expressed in ng cm^−2^, was obtained. The values reported in this work account for the various dilution and sample splitting steps in the extraction process but do not, however, account for the collection efficiency of the Nomex wipe, which is approximately 30% [33].

### 2.4. Screening of drugs by TD-DART-MS

Thermal desorption direct analysis in real time mass spectrometry (TD-DART-MS) was employed for the non-targeted screening analysis due the existence of in-house libraries that provide rapid searching capabilities of the resulting mass spectra. A 10 µL aliquot of the extract prepared for screening analysis was pipetted onto a PTFE-coated fiberglass wipe and analyzed by TD-DART-MS. The TD-DART-MS system used a JEOL AccuTOF JMS T100-LP time-of-flight mass spectrometer (JEOL USA, Peabody, MA) coupled with a DART ion source (IonSense, Saugus, MA) and an in-house built thermal desorption unit.[31] A thermal desorber temperature of 265 °C was utilized with a 400 °C DART gas temperature, a +100 V DART exit grid voltage, and zero-air nitrogen as the ionization gas. Mass spectrometer settings included operation in positive ionization mode, a 400 V peaks voltage, a +5 V orifice 2 and ring lens voltage, and a mass scan range of 60 *m*/*z*–700 *m*/ *z* at 1 s scan^−1^. Both molecular and fragmentation spectra were collected by using an orifice 1 voltage switch between +20 V and +60 V. Blank wipes were also analyzed in between samples to allow for background subtraction. PEG-600 was used as a mass calibrant and was analyzed before and after each batch of samples. The resulting mass spectra were searched against an in-house created library of over 300 narcotics and excipients for the characteristic molecular ions (in the +20 V spectra). The spectral search parameters included: identification of the protonated molecular ion of the compound at greater than 5% relative abundance within ± 5 amu of the calculated accurate mass.

### 2.5. Overview of samples collected

**Fig. 2:**
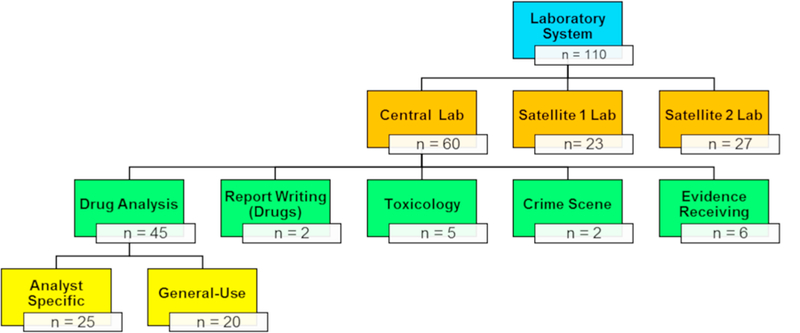
Breakdown of the locations where samples were collected throughout the laboratory system.

For this study, 110 samples were collected from three forensic laboratories (60 from the central laboratory and 50 from the two satellite laboratories) in addition to three blanks. Of the 60 samples taken from the central laboratory, 45 were taken from the drug unit and were nearly equally split between the analyst-specific surfaces/items (20) and general-use surfaces/items (25), as shown in Fig. 2. Sampling was primarily focused in the drug unit since it is the only area where analysts handle bulk powders – and therefore was where the highest surface concentrations were expected. Two samples were also collected from the report writing section of the drug unit in the central laboratory to determine if there was transfer of drugs from the laboratory space to an area where personal protective equipment (PPE) is not worn. Six samples were taken from the evidence receiving unit to evaluate surface levels in an area where packaged drug evidence is handled, but not opened, and PPE is typically not worn. This area was also of interest because it is where drug evidence is returned after being analyzed and resealed. If the amount of residue created from opening the case is high, there is an opportunity to transfer that to the evidence receiving unit via contamination of the exterior evidence packaging. Seven samples were taken from other units in the laboratory – five from the toxicology unit (located adjacent to the drug unit) and two from the crime scene unit. Benchtops, made of phenolic resin, were the most frequently sampled surface as they represented the largest surface area in the laboratory and it is where drug evidence is manipulated. Two other surfaces that were commonly sampled included balances, (specifically in the drug unit) and storage cabinets or containers in both the drug unit and evidence receiving unit. Less frequently sampled surfaces included: chemical hoods, door handles, keyboards, microscopes, telephones, and instruments (both GC/MS systems and Fourier transform infrared spectrometers (FTIR)).

### 2.6. Overview of cleaning procedures

Cleaning procedures within the laboratories were dependent on the analyst, unit, and surface that was being sampled. All analysts cleaned their workspaces (*i.e.* balances and benches) between each case worked and cleaned any tools or instruments (*i.e.* microscopes) between each item within a case when such tools or instruments were used. Benches were also covered with paper during casework to minimize direct contact of benches with the evidence. Cleaning entailed spraying the surface(s) with reagent grade methanol and wiping dry with a Kimwipe (Kimberly Clark Professional, Roswell, GA, USA). General-use spaces and spaces within the evidence receiving sections were cleaned on a less frequent basis, due to either lack of use or infrequent contact with drug evidence outside of packaging.

## 3. Results and discussion

### 3.1. Overview of surface levels of drugs

From the 60 samples that were collected, 15 of the 18 drugs targeted in the LC/MS/MS analysis were detected in at least one sample. Three drugs – acryl fentanyl, JWH-203, and 3,4-methylenedioxymethamphetamine (MDMA) were not detected in any sample and therefore are not reported in any subsequent figures and tables.

**Fig. 3:**
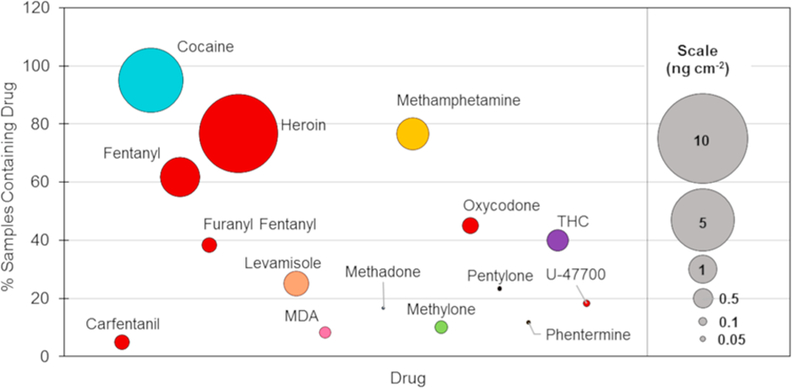
Bubble chart showing the relationship between the percentage of samples collected which contain a drug (y-axis) and the average amount collected (bubble size) at the main laboratory. Drugs are listed in alphabetical order across the x-axis. Drugs of similar structure are colored the same.

One way to visualize the results is to show the global averages of the surface level of drugs measured throughout the entire laboratory. Fig. 3 shows the average level of the quantified drugs (in ng cm^−2^) represented by the bubble size, with larger bubbles indicating a higher level of background. The overall prevalence of the drug is shown on the y-axis and drugs of a similar class are shown in the same color. This figure provides useful information for direct comparisons of the average surface level and overall prevalence of targeted substances within the lab and can be used to compare one laboratory to another.

The most commonly recovered drug in the central laboratory was cocaine (95% of surfaces), followed by heroin and methamphetamine (77% of surfaces each). A higher prevalence of these three drugs was expected given the number of cases containing these compounds, when compared to novel psychoactive substances (NPSs) like synthetic cathinones and fentanyl analogs. Case statistics for the year 2017 showed that 19% of all exhibits examined contained cocaine, while 13% contained heroin [35]. In addition to being frequently encountered, these substances were often received as powders, which aerosolize more easily when they are poured out to be weighed (compared to pills, liquids, and plant materials), resulting in a greater contribution to background concentrations than other forms of evidence. Fentanyl was the fourth most frequently detected drug in the laboratory (62% of surfaces), which correlated to the rapid rise in fentanyl-related cases throughout the country. A total of four synthetic opioids (fentanyl, furanyl fentanyl, carfentanil, and U-47700) were recovered off multiple surfaces within the laboratory, indicative of the expanding presence of opioids in casework.

The drug with the highest average surface level was heroin, followed by cocaine and fentanyl. The magnitude of the background was likely a combination of both the frequency with which the drug was encountered in casework and the drugs’ purity. The relative purity of the drug can be highlighted by comparing the relative background of heroin to fentanyl – which is typically present at a much lower percentage (5%–7% by weight) than heroin [36]. Many of the synthetic cathinones and fentanyl analogs were present on the surface at significantly lower levels (sub ng cm^−2^) and can likely be attributed to lower number of cases containing those samples. Similarly, the surface levels of THC were lower than cocaine and heroin, because it is commonly submitted as plant material which has a lower propensity to aerosolize and accumulate on surfaces.

**Fig. 4:**
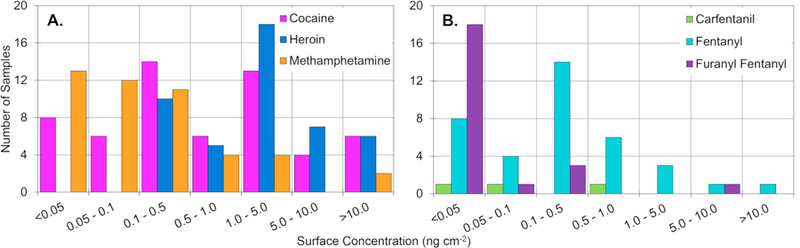
The distributions of surface concentrations of six drugs of interest from the central laboratory (n = 60). The distributions for the remaining drugs analyzed can be found in Fig. S1.

While the bubble chart in Fig. 3 provides a useful overview comparison of the drugs examined, it does not provide a representation of the range of levels detected, nor does it provide information to highlight differences within the various units of the laboratory. The range of surface levels encountered in the laboratory is shown in Table 2, Figs. 4, and S1. Table 2 provides the mean, median, and range of surface levels throughout the central laboratory. The range of surface concentrations varied quite significantly for the commonly encountered drugs (cocaine, heroin, methamphetamine, and fentanyl) and spanned up to four orders of magnitude for cocaine. Less frequently submitted drugs such as methylone and MDA, showed a tighter range, which could be attributed to a lower probability for surfaces with high background. Table 2 also provides a comparison of the average and median surface levels. For all drugs the median surface level was lower than the average, indicating the distribution for most of the drugs was skewed right, with the majority of samples having a surface concentration of less than 1 ng cm^−2^. This trend is shown in Figs. 4 and S1 and was found for all drugs except cocaine and heroin. Heroin exhibited a distribution that was skewed left while cocaine showed a roughly equal distribution across the concentration range.

**Fig. 5:**
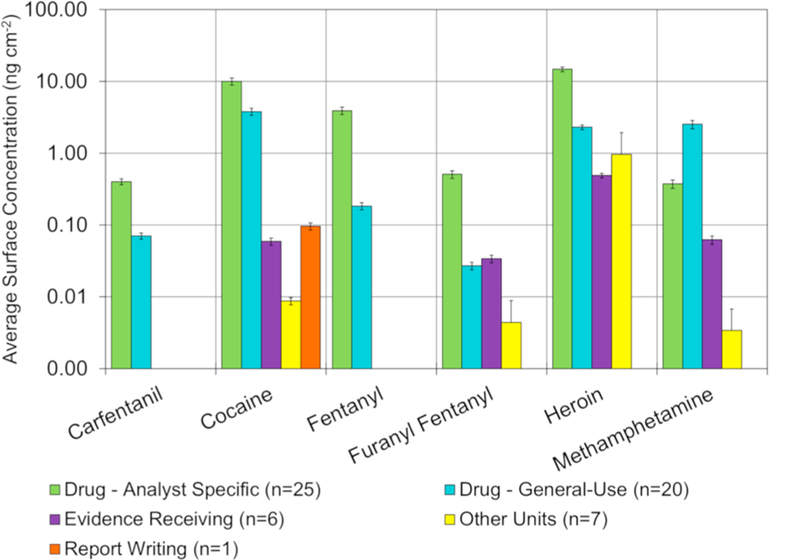
Comparison of surface concentrations between different locations within the central laboratory (n = 60). Error bars represent the measurement uncertainty. Note the y-axis is log-scale.

The location and surface or item type information was recorded for all samples to better investigate the variation of surface levels of drugs across the laboratory. Filtering the average surface level by the four units and the report writing area within the laboratory provides a clearer picture of the relative background levels of drugs throughout the laboratory. Of the 15 drugs detected by LC/MS/MS, 11 were found exclusively within the drug unit – only cocaine, heroin, methamphetamine, and furanyl fentanyl were found elsewhere. Fig. 5 (Tables S1 and S2) provides the average level of six select drugs collected in the five unit locations within the laboratory. The six drugs which are shown in this and subsequent figures, were chosen because they represent the four most commonly encountered drugs (cocaine, methamphetamine, heroin, and fentanyl) as well as two other hazardous synthetic opioids (furanyl fentanyl and carfentanil). In all cases the drug unit was either the only unit where the drug was detected or was the unit with the highest average surface concentrations. For the more commonly encountered drugs, such as heroin, the levels outside of the drug unit were at least an order of magnitude lower than the average level within the drug unit. The following sections will provide a more in-depth look at the results from the different locations within the laboratory.

### 3.2. Drug unit – analyst specific surfaces/items

**Fig. 6:**
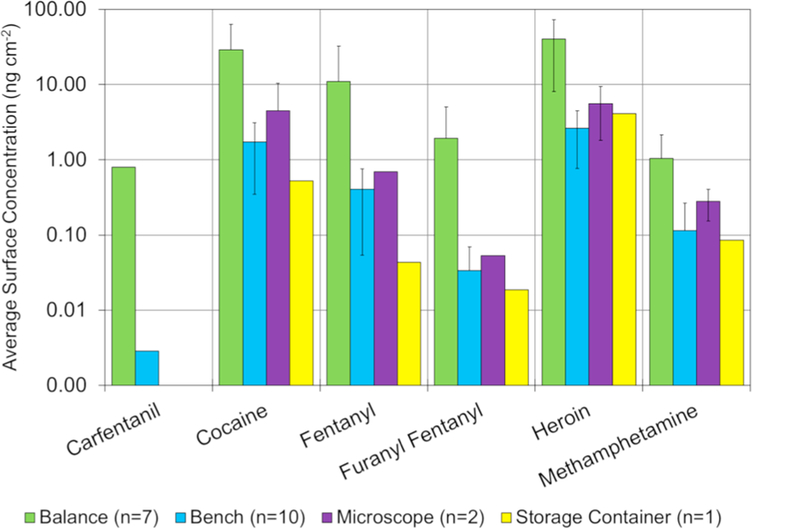
Average surface area levels of 6 of the 15 drugs broken down into the surface/item type from the analyst space in the central laboratory. Error bars represent the standard deviation of the measurement. The number of measurements for each surface is given in the legend. Note the y-axis is log-scale.

The samples collected within the drug unit were delineated between analyst specific surfaces and general-use surfaces. This delineation was made to study areas where the majority of the bulk handling of drug casework takes place (analyst specific surfaces) as opposed to areas where bulk material is not typically handled (general-use space). Within the analyst space, benchtops, balances, microscopes, and storage containers were sampled. Fig. 6 shows the average surface levels of the six selected drugs as a function of the various analyst specific surfaces or items sampled. The balances (Fig. 6 – green) contained the highest surface concentrations for most drugs examined. Levels on the benchtops were typically an order of magnitude lower than those recovered off balances. Higher concentrations of drugs on the balances may be due to repeated weighing of bulk powders, which presents the opportunity for static charge to build up within the balance and cause inadvertent spreading of the powder on and around the balance pan. Also, balances are not covered with a protective covering (*e.g.* paper, foil, sorbent material) that could be periodically removed. Balances are also more difficult to clean than benchtops, and cannot be sprayed down with the organic solvents in the manner that benches are commonly cleaned. The high levels collected off balances represents an opportunity for improvements in cleaning procedures to help lower the background. The distribution of drugs within the analyst space followed the overall trends within the laboratory, where heroin was the most prevalent and most abundant drug, followed by cocaine, methamphetamine and fentanyl.

Results also showed that the surface concentration of drugs on the microscope was higher than the bench. This was unexpected since the microscopes are typically only used for the analysis of plant materials. The higher levels may be a result of less frequent cleaning. The storage container sampled was used to store cases in progress and was found to have similar surface concentrations as the benches. The residue recovered from inside the storage container likely represents transfer from the exterior of the evidence containers – either from the time of collection or secondary contamination from when the case was opened. Storage containers may be useful to better understand to what extent the trace residues on the outside of packaging are transferred from evidence packaging to a secondary surface.

**Fig. 7:**
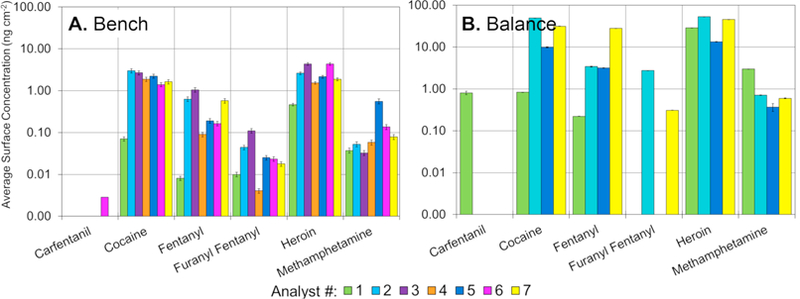
Surface levels of selected drugs found on benches (A.) and balances (B.) as a function of analyst in the central laboratory. Error bars represent the measurement uncertainty. Balances were not measured for analyst 3, 4, or 6. Note the y-axis is log-scale.

A further breakdown was completed to assess what, if any, differences exist across the benches and balances belonging to different analysts. Analyst spaces were randomly assigned a number and all samples collected from an individual’s space were noted with that sample number. Fig. 7 shows the six selected drugs as a function of analyst and surface type within the analyst space. It should be noted that the benchtop was sampled for all seven analysts, however, the balances were only sampled for four of the analysts. Differences in the surface concentration on the benches were significantly lower for cocaine and heroin (0.07 ng cm^−2^–3.00 ng cm^−2^, 52% RSD, and 0.47 ng cm^−2^–4.34 ng cm^−2^, 58% RSD, respectively) than the synthetic opioids, which all showed greater than 100% relative standard deviation. Differences of the balances was low for heroin as well (13.33 ng cm^−2^–53.35 ng cm^−2^, 51% RSD) and higher than 100% RSD for all remaining drugs. Interestingly, higher bench surface levels did not necessary correlate to higher balance levels. Since analyst numbers were randomized it was not possible to determine if the level of drugs on the benchtop and balances correlated to the analyst’s caseload. Variation in drug levels may be due to the differences in caseload or differences in cleaning procedures and/or cleaning frequency.

### 3.3. Drug unit – general-use surfaces/items

**Fig. 8:**
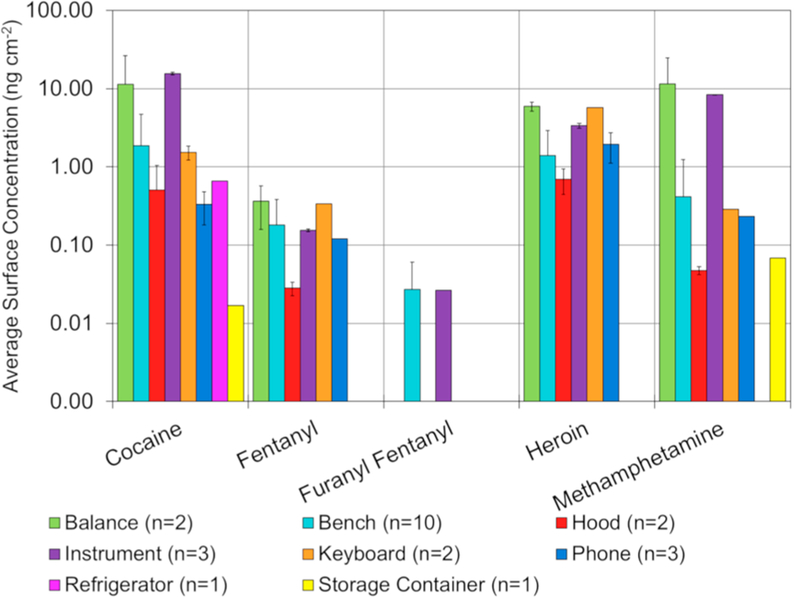
Average concentrations of 5 of the 15 drugs detected divided into the surface/item type from the general space in the central laboratory. Carfentanil was not found on any surface in the general space. Uncertainties represent the standard deviation of the measurement. Note the y-axis is log-scale.

While the analyst space in the drug unit is typically where bulk drugs are handled, it was important to also understand where else in the unit there was detectable levels of drugs. Because of this, samples were also collected across the general-use space within the unit, and focused predominantly on analytical instruments, balances, and benches, along with surfaces analysts may contact while not wearing PPE (such as refrigerators or telephones). Fig. 8 shows the average surface levels of the six selected drugs on different general use surfaces throughout the drug unit. The lesser degree to which general space balances (Fig. 8) were used for weighing powders was observed by comparing these levels with those from the analyst space (Fig. 6). While the analyst specific balances had average levels of cocaine and heroin of 28.76 ng cm^−2^ and 32.04 ng cm^−2^ respectively, the general-use balances had levels of 15.13 ng cm^−2^ and 0.75 ng cm^−2^. With the exception of methamphetamine, surface levels were less than half of the general space balances, compared to the analyst space samples. Surface levels throughout the entirety of the general space were typically lower, potentially indicating a minimal level of transfer throughout the laboratory. Still, most surfaces had detectable levels of methamphetamine, cocaine, and heroin.

The level of cocaine collected from the exterior of analytical instruments illustrated a potential area where additional cleaning procedures may need to be considered. The amount collected off the GC/ MS, where all case samples are in solution and no bulk powders are handled, was substantially lower (1.15 ng cm^−2^ and 4.51 ng cm^−2^) than the level collected off the FTIR (41.05 ng cm^−2^), where bulk powders are handled. Loading or cleaning of the powder off the diamond cell for analysis likely contributed to the higher levels on the FTIR surface. Additionally, cleaning the FTIR likely led to transfer onto the adjacent bench which had an elevated surface concentration of cocaine (2.02 ng cm^−2^) compared to the GC/MS bench (0.11 ng cm^−2^), and was roughly equivalent to the average amount of cocaine collected off the analyst benches (1.72 ng cm^−2^).

Surfaces that analysts are likely to encounter while not wearing PPE (*e.g.* telephones, refrigerators, etc.) had measurable concentrations of the major drugs (cocaine, fentanyl, heroin, and methamphetamine). The refrigerator handle used for the storage of standards was the cleanest of these surfaces (only containing cocaine), followed by the telephones, and then the keyboards for the instruments. Telephones located near analyst benches had noticeably higher levels than telephones located away from analyst benches (*i.e.* in the instrument room). For instance, there was 2.50 ng cm^−2^ of heroin on the telephone closest to the analyst benches compared to non-detectable levels of heroin on the phone next to the GC/MS. From a safety aspect, the considerably lower levels of opioids in the general space may provide confidence that transfer of particulate across the laboratory was minimal.

### 3.4. Evidence receiving and other units

Outside of the drug unit, the surface levels of drugs detected dropped substantially. While the analyst and general space within the drug unit had 14 of 18 and 15 of 18 compounds detected respectively, the evidence receiving unit had only 4 drugs present (cocaine, furanyl fentanyl, heroin, and methamphetamine). Countertops used for evidence intake (from the submitting agency) and distribution (to the analyst) were sampled to identify what, if any, residue is transferred into and/or out of the drug unit. Low levels (< 0.01 ng cm^−2^) of cocaine were detected on both benches, with no other drugs present at quantifiable levels. The lack of significant background within this area was important because evidence technicians do not typically wear the same level of personal protective equipment as drug chemists.

Also within the evidence receiving section, a number of bins and drawers used to store cases in the drug vault were sampled. The bins and drawers contained cocaine at higher levels (≤0.1 ng cm^−2^) than what was recovered off of the benches. This higher background was likely caused by build-up of residues from the exterior evidence packaging over time. Heroin and methamphetamine were also detected at levels less than or equal to 2 ng cm^−2^, and one drawer contained a trace amount (0.03 ng cm^−2^) of furanyl fentanyl. Comparison of the drawers used to store unprocessed evidence and processed evidence showed no significant difference in concentration.

In addition to the drug and evidence receiving units, five sample were collected from the toxicology unit and two from the crime scene unit to evaluate different environments throughout the laboratory. Much like the evidence receiving unit, only four substances (cocaine, furanyl fentanyl, heroin, and methamphetamine) were detected on the benches in the toxicology unit. Heroin was the only drug detected above 0.1 ng cm^−2^, where two benches had concentrations of 0.15 ng cm^−2^ and 0.24 ng cm^−2^. These levels were similar to the lowest level recovered off of a bench in the drug unit (0.26 ng cm^−2^). Levels of cocaine, furanyl fentanyl, and methamphetamine were all below 0.04 ng cm^−2^. It should be noted that the layout of the laboratory allowed for entry of drug analysts into the toxicology unit directly from the drug unit. This may pose a higher probability of transfer than to a unit that does not have direct access to the drug unit.

For the crime scene unit, a crime scene van and evidence storage locker were sampled and both contained a low level (≤0.01 ng cm^−2^) of cocaine. The van sample also contained a higher level of heroin (24.42 ng cm^−2^) which may be due to either the transportation of evidence containing heroin or the contamination of the floor of the van from technicians entering a contaminated scene. Further work is attempting to better understand the drug levels in a crime scene environment. Two samples were also collected from the report writing section for drug analysts and only cocaine was recovered, at a level of < 0.2 ng cm^−2^.

### 3.5. Using TD-DART-MS for non-targeted screening of additional compounds

In addition to the LC/MS/MS quantitation of all samples analyzed, a separate aliquot was used for non-targeted screening by TD-DART-MS to determine what additional compounds may be present on these surfaces. Due to the lack of a chromatographic step, TD-DART-MS is considered a presumptive technique, as other chemicals may produce the same ions. The TD-DART-MS library used contains over 300 different drugs, excipients, and cutting agents. While the search list did include the 18 drugs that were part of the LC/MS/MS analysis, they were not reported in this section. Only the (+20 V) low voltage spectra was searched.

A total of 24 additional compounds were presumptively identified in the 60 samples that were analyzed from the central lab. The three most commonly identified compounds were quinine (a cutting agent present in 20 samples), atropine (a cutting agent present in 18 samples), and JWH-73 (a synthetic cannabinoid present in 9 samples). Three additional synthetic opioids, acetyl or benzyl fentanyl, cyclopropyl fentanyl, and methoxybutyryl fentanyl were detected in at least one sample from the drug unit. The majority of the 24 additional compounds identified (17 of 24) were only present in 3 or less samples. Table S3 provides a listing of all additional compounds that were identified by TD-DART-MS.

### 3.6. Comparison across multiple laboratories within the laboratory system

While the data presented thus far has been specific to the central laboratory, additional samples were also taken from the drug units (analyst specific space and general-use space) and evidence receiving areas of the two satellite laboratories. This contributed an additional 50 samples (23 from satellite 1 laboratory and 27 from satellite 2) to the total sample set. The satellite laboratories were studied to identify whether significant differences were observed and could be attributed to factors such as casework, environments, or differences in cleaning procedures.

The general trends (Tables S4 and S5 and Figs. S2–S4) between the laboratories were similar in that cocaine and heroin were the most abundant and frequently encountered drugs throughout the lab. Satellite laboratory 2 had slightly higher cocaine levels, which may be attributable to the higher percentage of exhibits containing cocaine that lab analyzes (55% of positive exhibits contained cocaine compared to 42% and 40% in the central laboratory and the satellite 1 laboratory, respectively). Differences in heroin and opioid background did not appear, however, to track with percentage of positive exhibits. While the percentage of exhibits containing both heroin and the opioids was highest at satellite laboratory 1, (33.5% of heroin and 8.5% fentanyl or furanyl fentanyl in all the positive exhibits analyzed in 2017) the number of cases was highest at the central laboratory (1138 heroin exhibits and 255 fentanyl or furanyl fentanyl exhibits) which was more than both of the satellite laboratories combined. Because of these differences, it is unclear what factor, if any, drives the overall surface concentrations. Likely it is a combination of several factors including the frequency of a drug’s occurrence, the amount of drug received, the laboratory infrastructure, and the cleaning procedures of individual analysts. Other unique differences that were observed between the laboratories included noticeably higher levels of oxycodone in satellite laboratory 1 (even though all three laboratories had approximately 10% of positive exhibits containing oxycodone), and higher levels of phentermine in the satellite laboratory 2.

**Fig. 9:**
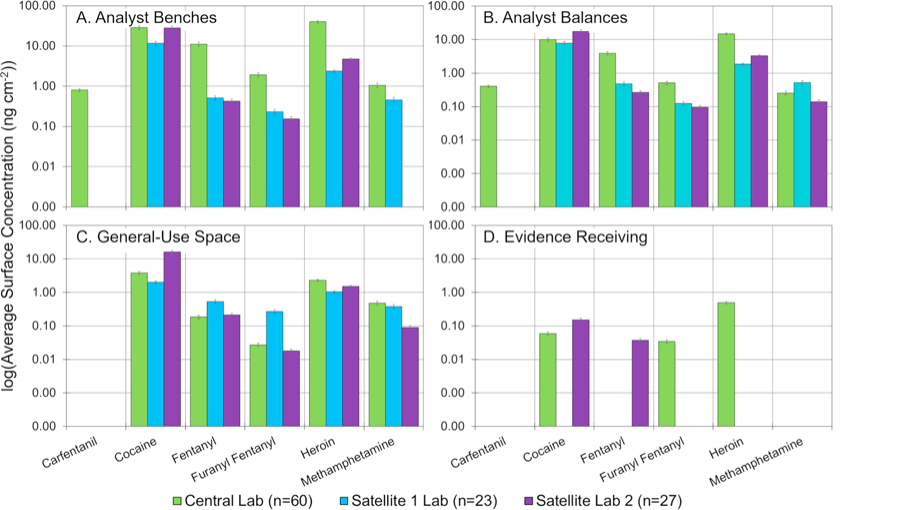
Comparison of surface concentrations of the central laboratory and two satellite laboratories in terms of (A.) analyst benches, (B.) analyst balances, (C.) general-use space, and (D.) evidence receiving. Error bars represent the measurement uncertainty. Note the y-axis is log-scale.

A breakdown of different locations throughout all three laboratories is shown in Fig. 9. In most instances, surface concentrations of similar surfaces (*i.e.*, benches or balances) were within the same order of magnitude across the three laboratories. There were two notable deviations from this general trend – opioids in the analyst space and the evidence receiving areas. The analyst spaces in the central lab had noticeably higher levels of heroin and the synthetic opioids than the two satellite laboratories. Additionally, balances within the central lab averaged twenty times higher in concentration than the satellites (which had approximately the same surface concentration). The exact cause of this is not fully understood, given the percentage of exhibits containing those drugs is not highest at the central laboratory, indicating other factors, such as differences in cleaning protocols or cleaning frequencies. These results correlated to the analyst bench measurements though the differences were less pronounced. Interestingly as well was the absence of methamphetamine from analyst surfaces in the satellite 2 laboratory even though roughly 3% of positive exhibits contained the drug. Differences in evidence receiving areas were also pronounced. The evidence receiving area at satellite 1 laboratory had one drug present at detectable levels while the satellite 2 laboratory had ten (Tables S4 and S5). This was likely due to differences in countertop material (epoxy versus wood). The laboratory with the wood evidence receiving desk likely had trace levels of drugs absorbed into the porous wood during cleaning.

Results of the screening using TD-DART-MS (Table S6) showed several cutting agents and drugs were present on various surfaces throughout the laboratory. No search hits were produced for any additional fentanyl analogs or other synthetic opioids in the satellite labs. Detection of carfentanil was limited to the central lab, where bulk quantities were analyzed, even though one of the satellite labs did have several cases of that drug.

## 4. Conclusion

Past studies show that public spaces, currency, and wastewater all have detectable environmental background levels of drugs. Certain operational environments such as police stations have increased background levels due to the seizure and packaging of suspected drugs after arrests. Similarly, this work shows that the processing and handling of seized-drug cases within a forensic laboratory results in potential introduction of trace levels of drugs being deposited onto surfaces. For this work, a robust sampling procedure and quantitative method was employed to measure the surface levels of drugs in a forensic laboratory at a single point in time. The highest surface concentrations were measured on balances located at analyst desks. Surface levels on benches were found to be approximately an order of magnitude lower, and can likely be attributed to increased cleaning and the ability to cover benches with a protective surface. The bulk of the background was found to be cocaine, heroin, fentanyl, and methamphetamine, which corresponded to the most frequently encountered drugs in case samples. Other drugs were encountered on various surfaces on a less frequent basis. As expected, concentrations were highest at analystspecific space, and typically dropped significantly in the general use areas. Background was minimal in the evidence receiving area, which was encouraging given the lower level of PPE worn by evidence technicians. Drug residues were also recovered in the toxicology unit and crime scene units, but typically at levels below those observed in the drug unit. Differences between laboratories were observed, especially when the average surface concentrations of opioids were compared. These differences could be attributed to the higher relative frequency with which the main laboratory received opioid cases. Trends between analyst specific space, general-use space, and evidence receiving were largely similar.

By measuring the background levels of drugs in the laboratory, a number of things can be evaluated to improve processes in the laboratory. For instance, areas where higher levels are observed can be noted and designated for a more thorough or more frequent cleaning. Additionally, as new housekeeping policies are implemented, such as changes in sample handling or cleaning, repeat measurements can be made to quantify the effects of such changes. Likewise, results like these can help quality managers identify and modify workflow processes that most contribute to the background. They can also demonstrate whether engineering controls that are in place are minimizing the spread of substances throughout a laboratory. The persistence of trace residues and the efficacy of the cleaning protocols are currently under investigation and can be evaluated using this platform. Future publications will discuss whether the measured surface levels are detectable using current casework equipment as well as the implications of those values. In addition, ongoing collaborations across government agencies aim to address the interpretation of the levels from an occupational safety and health perspective and whether enhanced PPE can help safeguard personnel involved in the handling and processing of evidence.

## Supplementary Material

Supp1

## Figures and Tables

**Table 1:** Relevant LC/MS/MS information including retention time, transitions, limits of quantitation, and measurement uncertainty. The MS/MS transition in bold was the transition used for quantitation. Measurement uncertainty represents the average deviation between the known concentration of the calibration curve verification (CCV) samples and their measured concentrations across the calibration range.

Analyte	Retention Time (min)	MS Transitions		LOQ (µg wipe^−1^)	Measurement Uncertainty	Internal Standard
		Q1	Q3			
Acryl Fentanyl	8.5	**335**	**188**	0.01	± 13.7%	Fentanyl-d_5_
		335	105			
Carfentanil	8.6	**395**	**113**	0.01	± 9.6%	Fentanyl-d_5_
		395	134			
Cocaine	7.6	**304**	**182**	0.01	± 11.4%	Cocaine-d_3_
		304	105			
Fentanyl	8.5	**337**	**188**	0.05	± 11.9%	Fentanyl-d_5_
		337	105			
Furanyl Fentanyl	8.8	**375**	**188**	0.01	± 12.3%	Fentanyl-d_5_
		375	105			
Heroin	7.3	**370**	**328**	0.025	± 7.1%	Heroin-d_9_
		370	310			
JWH-203	12.8	**340**	**125**	0.025	± 10.9%	THC-d_9_
		340	214			
Levamisole	6.5	**205**	**128**	0.1	± 10.2%	Cocaine-d_3_
		205	91			
MDA	5.9	**180**	**135**	0.05	± 9.8%	Meth-d_5_
		180	77			
MDMA	6.3	**194**	**77**	0.025	± 11.4%	Meth-d_5_
		194	135			
Methadone	10.0	**310**	**265**	0.01	± 10.1%	THC-d_9_
		310	105			
Methamphetamine	6.1	**150**	**119**	0.1	± 13.6%	Meth-d_5_
		150	91			
Methylone	6.1	**208**	**117**	0.05	± 8.4%	Cocaine-d_3_
		208	132			
Oxycodone	6.1	**316**	**241**	0.01	± 9.3%	Cocaine-d_3_
		316	212			
Pentylone	7.2	**236**	**131**	0.01	± 7.1%	Cocaine-d_3_
		236	174			
Phentermine	6.1	**150**	**133**	0.1	± 9.3%	Meth-d_5_
		150	91			
THC	12.3	**315**	**193**	0.025	± 10.0%	THC-d_9_
		315	123			
U-47700	8.1	**329**	**173**	0.025	± 7.1%	Fentanyl-d_5_
		329	81			

**Table 2:** Overall summary of the mass of drugs recovered from samples in the central laboratory normalized to ng cm^−2^. Also reported is the range of actual amounts of material collected and a comparison of the results to those by Doran et al.

Drug	% Samples Containing Drug	Mean Surface Area Level (ng cm^−2^)	Median Surface Area Level (ng cm^−2^)	Range of Levels (ng cm^−2^)	Range of Levels (μg wipe^−1^)	Highest Level Reported by Doran et al. [25] (pg wipe^−1^)
Carfentanil	7	0.292	0.037	0.003–0.802	0.01–0.51	xx
Cocaine	95	5.215	0.502	0.002–88.303	0.01–56.51	71.43
Fentanyl	62	2.008	0.204	0.008–54.968	0.04–35.18	xx
Furanyl Fentanyl	38	0.280	0.003	0.004–5.487	0.01–3.51	xx
Heroin	77	7.750	1.965	0.152–97.255	0.63–62.25	xx
Levamisole	25	0.808	0.228	0.051–4.276	0.12–2.74	xx
MDA	8	0.182	0.170	0.019–0.417	0.06–1.28	xx
Methadone	18	0.014	0.008	0.002–0.038	0.01–0.09	0.27
Methamphetamine	77	1.320	0.092	0.021–23.583	0.10–22.71	326.16
Methylone	10	0.222	0.141	0.024–0.568	0.02–0.48	xx
Oxycodone	45	0.347	0.139	0.014–3.251	0.05–2.08	0.84
Pentylone	23	0.032	0.016	0.004–0.192	0.01–0.08	xx
Phentermine	17	0.027	0.016	0.009–0.086	0.02–0.10	xx
THC	38	0.620	0.435	0.048–2.841	0.25–2.21	1.95
U-47700	18	0.062	0.019	0.001–0.339	0.34–18.33	xx

## HIGHLIGHTS

- Surface concentration of drugs in a forensic lab was measured using LC/MS/MS and TD-DART-MS.
- Concentrations were highest in the drug chemistry section.
- Balances contained highest concentrations, showing the difficulty in cleaning them.
- Data can help address data integrity, safety and the effects of procedural changes.
