# Supplementary material for: A snapshot of drug background levels on surfaces in a forensic laboratory
Source: Forensic Chem. Author manuscript; Available in PMC 2019 Sep 25. (PMC6760002; doi:10.1016/j.forc.2018.09.001)
Supplement: Supp1 [file NIHMS1520844-supplement-Supp1.docx]

**Supplemental Information:**

**A Snapshot of Drug Background Levels on Surfaces in a Forensic Laboratory**

^a^Edward Sisco*, ^a^Marcela Najarro, ^b^Amber Burns

^a^National Institute of Standards and Technology, Gaithersburg, MD 20899

^b^Maryland State Police, Forensic Science Division, Pikesville, MD 21208

*edward.sisco@nist.gov, 301-975-2093

**Figure S1.** The distributions of surface concentrations of the remaining drugs of interest.


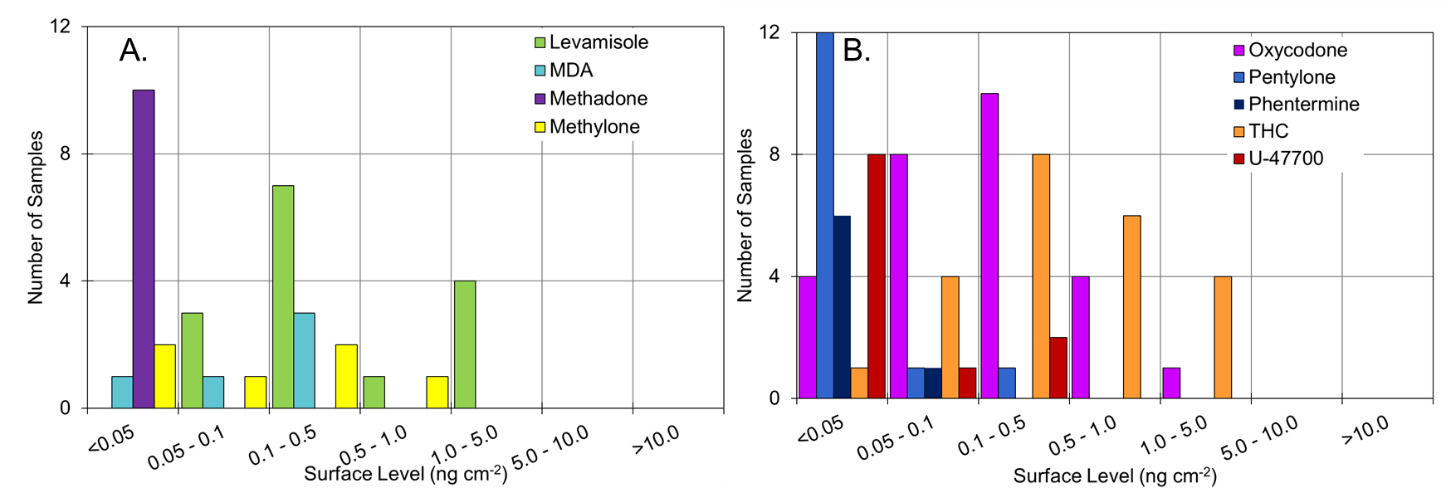


**Table S1.** Average background level of drug recovered as a function of location within the central laboratory. (An “x” indicates no samples in this location contained the drug). Uncertainties expressed are the measurement uncertainty. These values are normalized to unit area.

| **Drug** | **Average Background Level (ng cm^-2^)** | | | | |
| --- | --- | --- | --- | --- | --- |
|  | **Drug – Analyst Specific** | **Drug – General Use** | **Evidence Receiving** | **Other Units (Tox & Crime Scene)** | **Report Writing** |
| Carfentanil | 0.40 (±0.04) | 0.07 (±0.01) | x | x | x |
| Cocaine | 10.05 (±1.15) | 3.82 (±0.46) | 0.06 (±0.01) | 0.01 (±0.001) | 2.00 (±0.23) |
| Fentanyl | 3.93 (±0.47) | 0.18 (±0.02) | x | x | x |
| Furanyl Fentanyl | 0.51 (±0.06) | 0.03 (±0.003) | 0.03 (±0.004) | 0.04 (±0.004) | x |
| Heroin | 14.83 (±1.05) | 2.30 (±0.16) | 0.49 (±0.03) | 13.66 (±0.97) | x |
| Levamisole | 1.06 (±0.11) | 0.43 (±0.04) | x | x | x |
| MDA | 0.11 (±0.01) | 0.23 (±0.02) | x | x | x |
| Methadone | 0.02 (±0.002) | 0.01 (±0.001) | x | x | x |
| Methamphetamine | 0.38 (±0.05) | 2.54 (±0.35) | 0.06 (±0.008) | 0.03 (±0.003) | x |
| Methylone | x | 0.22 (±0.02) | x | x | x |
| Oxycodone | 0.25 (±0.02) | 0.48 (±0.04) | x | x | x |
| Pentylone | 0.02 (±0.001) | 0.04 (±0.003) | x | x | x |
| Phentermine | 0.03 (±0.003) | 0.02 (±0.002) | x | x | x |
| THC | 0.75 (±0.07) | 0.50 (±0.05) | x | x | x |
| U-47700 | 0.10 (±0.01) | 0.01 (±0.001) | x | x | x |

**Table S2.** Average mass of drug recovered as a function of location within the central laboratory. (An “x” indicates no samples in this location contained the drug). Uncertainties expressed are the measurement uncertainty. These values are not normalized to unit area.

| **Drug** | **Average Mass (µg swipe^-1^)** | | | | |
| --- | --- | --- | --- | --- | --- |
|  | **Drug – Analyst Specific** | **Drug – General Use** | **Evidence Receiving** | **Other Units (Tox & Crime Scene)** | **Report Writing** |
| Carfentanil | 0.26 (±0.02) | 0.09 (±0.01) | x | x | x |
| Cocaine | 9.09 (±1.04) | 5.13 (±0.58) | 0.12 (±0.01) | 0.03 (±0.003) | 0.05 (±0.005) |
| Fentanyl | 3.12 (±0.37) | 0.51 (±0.61) | x | x | x |
| Furanyl Fentanyl | 0.39 (±0.05) | 0.09(±0.01) | 0.07 (±0.01) | 0.08 (±0.01) | x |
| Heroin | 13.63 (±0.97) | 4.95 (±0.35) | 0.81 (±0.058) | 16.45 (±1.17) | x |
| Levamisole | 1.07 (±0.11) | 0.80 (±0.08) | x | x | x |
| MDA | 0.16 (±0.02) | 0.53 (±0.05) | x | x | x |
| Methadone | 0.03 (±0.003) | 0.03 (±0.003) | x | x | x |
| Methamphetamine | 0.39 (±0.05) | 2.87 (±0.39) | 0.11 (±0.02) | 0.12 (±0.02) | x |
| Methylone | x | 0.24 (±0.02) | x | x | x |
| Oxycodone | 0.32 (±0.03) | 0.76 (±0.07) | x | x | x |
| Pentylone | 0.04 (±0.003) | 0.03 (±0.003) | x | x | x |
| Phentermine | 0.05 (±0.005) | 0.08 (±0.008) | x | x | x |
| THC | 0.81 (±0.08) | 0.93 (±0.09) | x | x | x |
| U-47700 | 0.07 (±0.005) | 0.14 (±0.01) | x | x | x |

**Table S3.** Compounds presumptively identified by TD-DART-MS analysis of samples from the central laboratory. No additional compounds were identified in the evidence receiving section, other units, or the report writing area. (An “x” indicates no samples in this location contained the compound).

| **Drug** | **# Samples Identified** | | |
| --- | --- | --- | --- |
|  | **Drug - Analyst Specific** | **Drug – General Use** | **Other Units** |
| 2-MMC / 4-MMC | x | 1 | x |
| Acetylsalicylic Acid | 1 | 1 | x |
| Acetaminophen | 3 | 3 | x |
| Acetyl / Benzyl Fentanyl | x | 1 | x |
| Alprazolam | x | 1 | x |
| AMB-FUBINACA | 3 | x | x |
| Atropine | 11 | 5 | x |
| Butylone | 1 | 1 | x |
| Caffeine | 1 | 1 | x |
| Cannabinol | x | 2 | x |
| Cyclopropyl Fentanyl | x | 1 | x |
| Etizolam | 1 | x | x |
| HU-331 | 1 | 1 | x |
| JWH-018 | x | 1 | x |
| JWH-081 | 1 | x | x |
| JWH-073 | 5 | 4 | x |
| Lidocaine | x | 4 | x |
| MDPV | x | 1 | x |
| Mescaline | 1 | 3 | 1 |
| Methoxybutyryl Fentanyl | x | 1 | x |
| PCP | 3 | 3 | x |
| Phenacetine | 1 | 2 | x |
| Phenobarbital | x | 1 | x |
| Quinine | 10 | 10 | x |


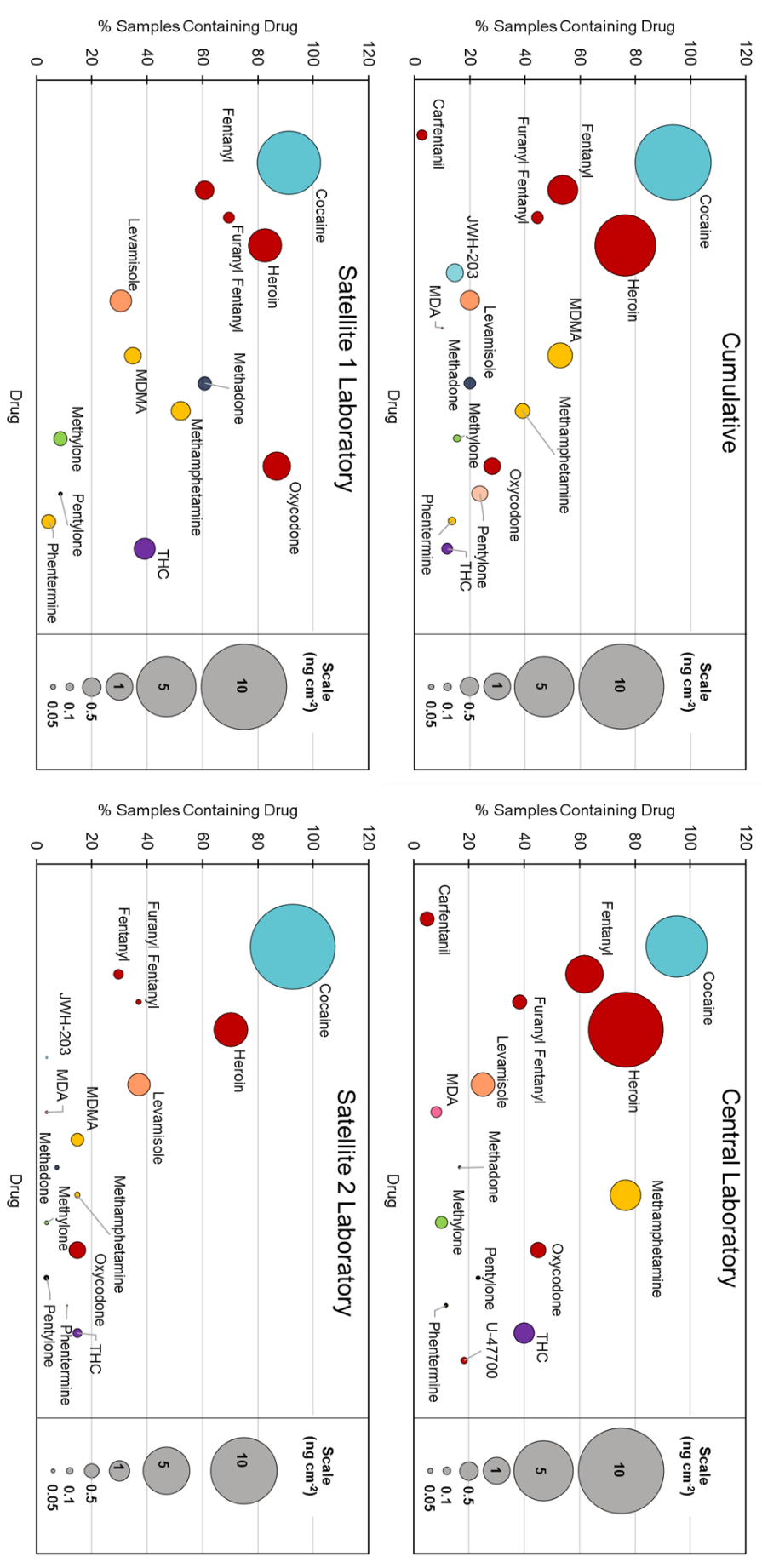


**Figure S2.** Bubble charts for the cumulative of the three laboratories examined and each of the individual laboratories examined. Drugs are listed in alphabetical order.

**
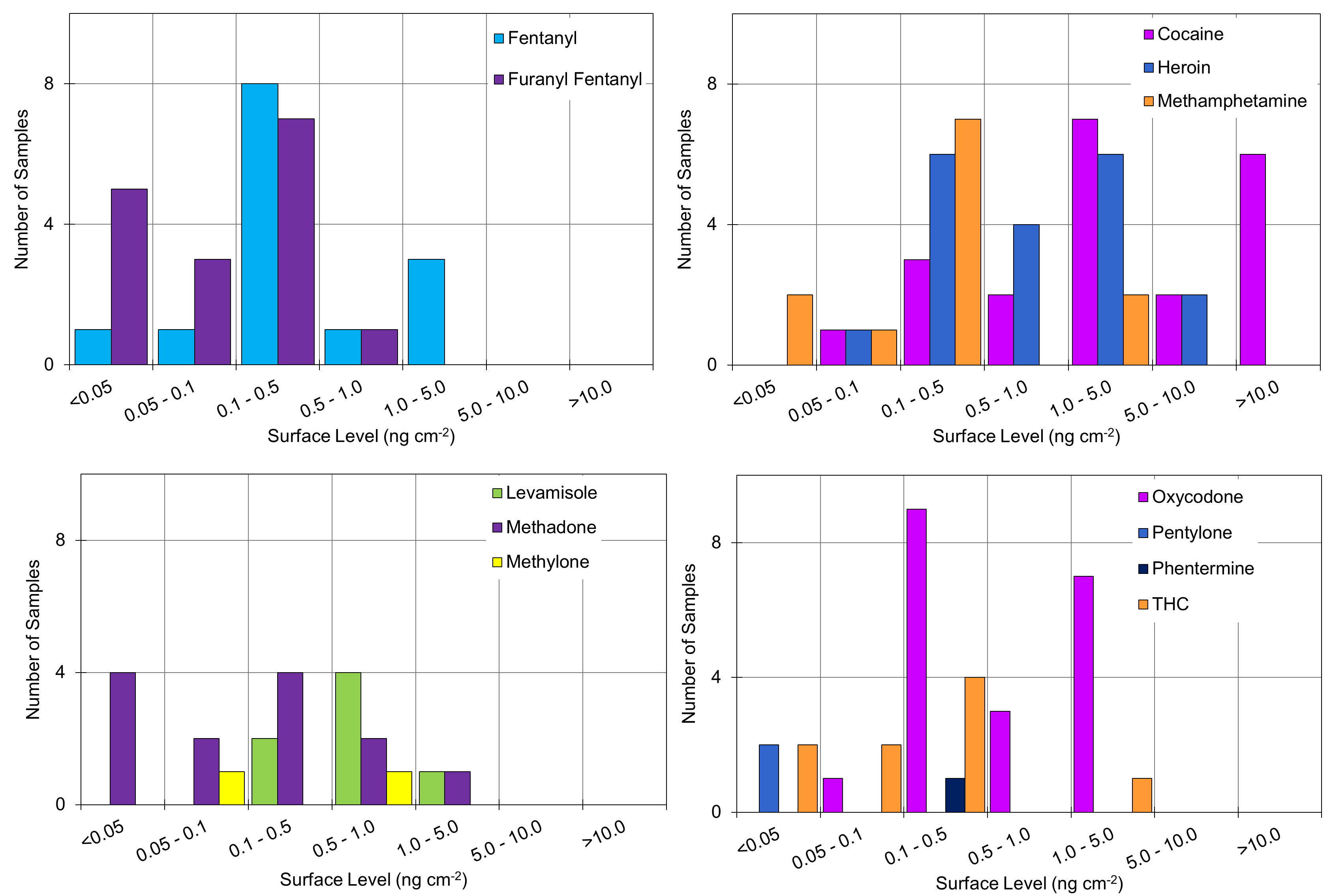
**

**Figure S3.** The distributions of surface concentrations of the quantified drugs for Satellite Laboratory 1. A total of 23 samples were taken from this laboratory.


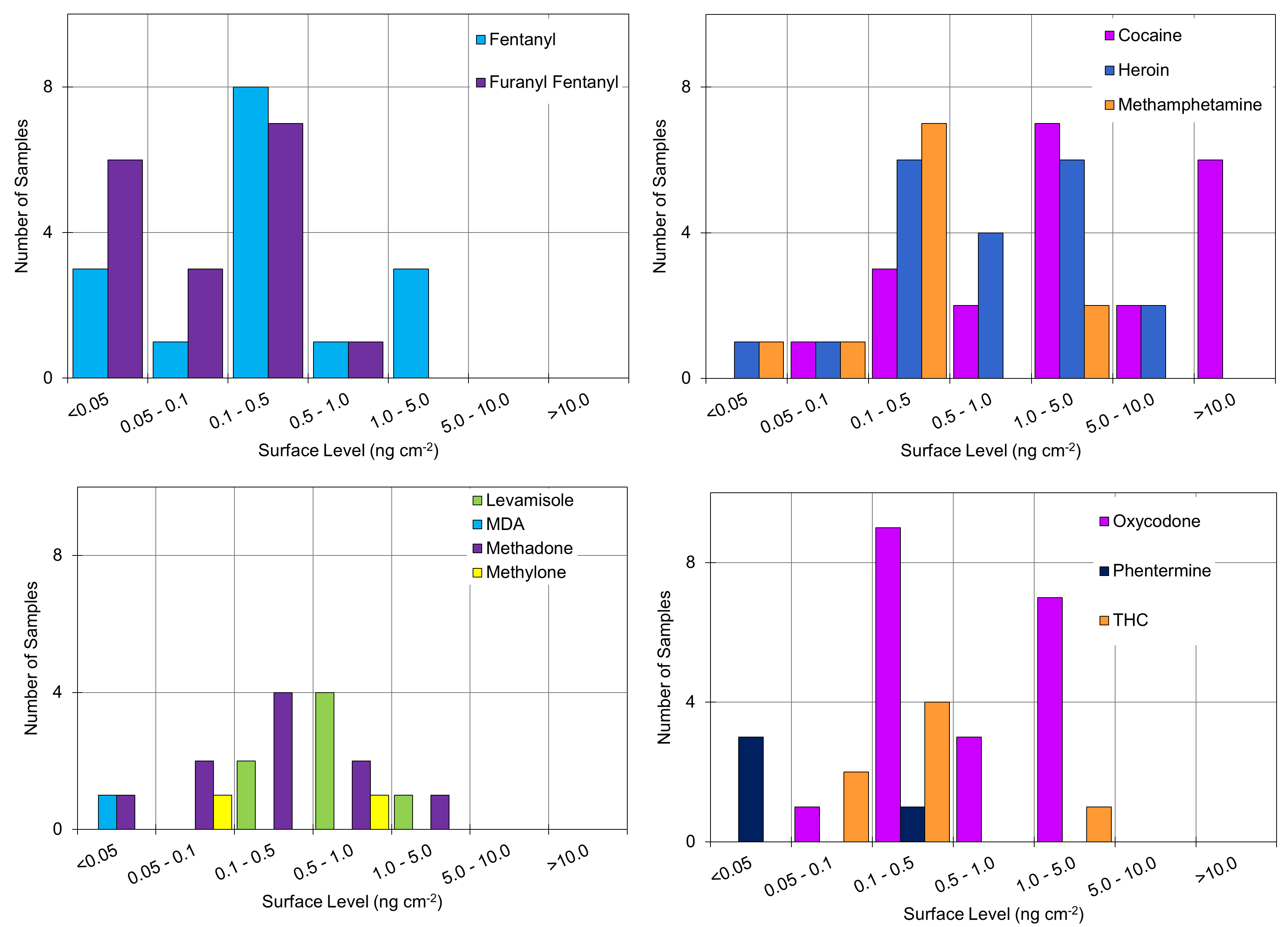


**Figure S4.** The distributions of surface concentrations of the quantified drugs for Satellite Laboratory 2. A total of 27 samples were taken from this laboratory.

**Table S4.** Average surface level of drug recovered as a function of location within the Satellite 1 laboratory. (An “x” indicates no samples in this location contained the drug). Uncertainties expressed are the measurement uncertainty.

| **Drug** | **Average Background Level (ng cm^-2^)** | | |
| --- | --- | --- | --- |
|  | **Drug – Analyst Specific (n=13)** | **Drug – General Use (n=8)** | **Evidence Receiving (n=2)** |
| Carfentanil | x | x | x |
| Cocaine | 7.86 (±0.90) | 2.01 (±0.23) | x |
| Fentanyl | 0.48 (±0.06) | 0.53 (±0.06) | x |
| Furanyl Fentanyl | 0.12 (±0.01) | 0.27 (±0.03) | x |
| Heroin | 1.85 (±0.13) | 1.03 (±0.07) | x |
| Levamisole | 0.80 (±0.08) | 0.40 (±0.04) | x |
| MDA | x | X | x |
| MDMA | 0.50 (±0.07) | 0.11 (±0.01) | x |
| Methadone | 0.32 (±0.03) | 0.06 (±0.005) | 0.01 (±0.001) |
| Methamphetamine | 0.52 (±0.04) | 0.38 (±0.04) | x |
| Methylone | 0.29 (±0.02) | x | x |
| Oxycodone | 1.51 (±0.14) | 0.34 (±0.03) | x |
| Pentylone | 0.03 (±0.002) | x | x |
| Phentermine | x | 0.29 (±0.03) | x |
| THC | 0.71 (±0.05) | 0.11 (±0.008) | x |
| U-47700 | x | x | x |

**Table S5.** Average surface level of drug recovered as a function of location within the Satellite 2 laboratory. (An “x” indicates no samples in this location contained the drug). Uncertainties expressed are the measurement uncertainty.

| **Drug** | **Average Background Level (ng cm^-2^)** | | |
| --- | --- | --- | --- |
|  | **Drug – Analyst Specific (n=15)** | **Drug – General Use (n=10)** | **Evidence Receiving (n=1)** |
| Carfentanil | x | x | x |
| Cocaine | 17.46 (±1.99) | 15.99 (±1.82) | 0.15 (±0.02) |
| Fentanyl | 0.26 (±0.03) | 0.21 (±0.03) | 0.04 (±0.004) |
| Furanyl Fentanyl | 0.10 (±0.01) | 0.02 (±0.002) | x |
| Heroin | 3.27 (±0.23) | 1.52 (±0.11) | x |
| Levamisole | 1.43 (±0.15) | 1.01 (±0.10) | 0.17 (±0.02) |
| MDA | x | x | 0.02 (±0.001) |
| MDMA | x | 0.51 (±0.05) | 0.02 (±0.002) |
| Methadone | x | 0.07 (±0.009) | 0.03 (±0.005) |
| Methamphetamine | 0.14 (±0.02) | 0.09 (±0.008) | x |
| Methylone | x | x | 0.05 (±0.005) |
| Oxycodone | 0.83 (±0.06) | x | 0.10 (±0.007) |
| Pentylone | x | 0.07 (±0.006) | 0.07 (±0.006) |
| Phentermine | x | 0.01 (±0.001) | 0.01 (±0.001) |
| THC | 0.24 (±0.02) | 0.09 (±0.006) | x |
| U-47700 | x | x | x |

**Table S6.** Compounds presumptively identified by TD-DART-MS analysis for the Satellite 1 Laboratory and Satellite 2 Laboratory. No additional compounds were identified in the evidence receiving section.

| **Drug** | **# Samples Identified** | | | |
| --- | --- | --- | --- | --- |
|  | **Drug - Analyst Specific** | | **Drug - General Use** | |
| **Laboratory** | **Sat 1** | **Sat 2** | **Sat 1** | **Sat 2** |
| 2-MMC / 4-MMC | 1 | 3 | x | x |
| Acetylsalicylic Acid | x | 2 | x | x |
| Acetaminophen | x | 1 | x | x |
| Amphetamine | x | 1 | x | x |
| Atropine | 1 | 1 | 3 | x |
| Butylone | x | 1 | x | x |
| Ephedrine | x | 4 | x | 1 |
| JWH-073 | 1 | x | x | x |
| Ketamine | 1 | x | x | x |
| MDPV | 1 | x | 2 | x |
| Methyl Phenidate | x | x | 4 | x |
| PCP | 1 | x | 2 | x |
| Phenacetine | x | 1 | x | x |
| Procaine | x | 1 | x | x |
| Quinine | 6 | x | 3 | x |
